# Prefabricated foot orthoses compared to a placebo intervention for the treatment of chronic nonspecific low back pain: a study protocol for a randomised controlled trial

**DOI:** 10.1186/s13047-018-0299-5

**Published:** 2018-10-16

**Authors:** Sean Sadler, Martin Spink, Samuel Cassidy, Vivienne Chuter

**Affiliations:** Discipline of Podiatry, University of Newcastle, 10 Chittaway Road, Ourimbah, NSW 2258 Australia

**Keywords:** Randomised controlled trial, Prefabricated foot orthoses, Chronic nonspecific low back pain, Gluteus medius, Transversus abdominis, Foot type

## Abstract

**Background:**

Prefabricated foot orthoses are used to treat chronic nonspecific low back pain, however their effectiveness and potential mechanism of action is unclear. The primary aims of the study are to investigate the effectiveness of prefabricated foot orthotic devices for reducing pain and improving function in people with chronic nonspecific low back pain over 52 weeks.

**Methods:**

This study is a participant and assessor blinded, parallel-group, superiority randomised (1:1) controlled trial. The study will recruit 60 participants aged 18 to 65 years with chronic nonspecific low back pain. Participants will undergo randomisation to a control group (The Back Book) or an intervention group (prefabricated foot orthoses and The Back Book). The primary outcome measures will be change in pain and function from baseline to 12 (primary time point), 26, and 52 weeks. Secondary outcome measures include: gluteus medius muscle activity and transversus abdominis muscle thickness from baseline to 12 weeks, physical activity over 12, 26, and 52 weeks, and correlation between foot type and change in measures of pain and function. Number of hours per day and week that the prefabricated orthoses are worn, as well as, adverse events will be self-reported by participants. Data will be analysed using the intention-to-treat principle.

**Discussion:**

This trial will primarily evaluate the effectiveness of prefabricated foot orthotic devices for reducing pain and improving function in people with chronic nonspecific low back pain over 52 weeks. It is expected that this study will provide clinicians and researchers with an understanding of the role that prefabricated foot orthoses may have in the treatment of chronic nonspecific low back pain and a potential mechanism of action, and whether foot type influences the outcome.

**Trial registration:**

ACTRN12618001298202.

**Electronic supplementary material:**

The online version of this article (10.1186/s13047-018-0299-5) contains supplementary material, which is available to authorized users.

## Background

Globally, low back pain (LBP) is the greatest cause of disability and is one of the major contributors to disease burden [1]. Direct costs of back pain have been estimated to be over AU$1 billion, most of which are associated with medical treatment [2]. Further to this, an additional AU$8.15 billion is lost through reduced earnings and decreased productivity both at work and home [2]. Recurrence rates of LBP are high, with up to 44% of LBP sufferers experiencing a return of symptoms within twelve months, and 85% having a recurrence over their life-time [3].

The aetiology of LBP is multifactorial with individual characteristics, psychosocial factors, and occupational demands linked to the development of the condition [3, 4]. Due to the varied nature of LBP, up to 85% of cases are classified as nonspecific [5], with the majority of these lasting for periods of greater than 3 months [6]. In such cases the LBP is referred to as chronic nonspecific LBP. Chronic nonspecific LBP is associated with high levels of pain, disability, depression, and reduced quality of life [7]. The evidence for the extensive range of chronic nonspecific LBP treatments has mixed levels of quality and shows highly variable outcomes [8–10].

Foot orthoses are one of the many treatment options available for chronic nonspecific LBP. The justification for the use of foot orthoses to treat chronic nonspecific LBP is based on several theoretical therapeutic mechanisms. These include reducing excessive or abnormal foot pronation [11, 12], achieving realignment of more proximal structures of the lumbopelvic-hip complex through adjustment of pronated foot posture [13, 14], and to increase the contact area of cavus feet so that shock absorption is improved [14, 15]. However, despite widespread use of this form of therapy, the evidence for the efficacy of this treatment for reducing pain and improving function in chronic nonspecific LBP suffers is limited.

A recent systematic review and meta-analysis reported that there are a lack of high quality trials investigating the use of foot orthoses to treat chronic nonspecific LBP [16]. Overall, the trials included in the systematic review were of moderate methodological quality, and had high heterogeneity, potentially masking the true extent of the beneficial effects of foot orthoses. Further to this, most of these trials used customised orthoses that were modified for individual participants [17–20], and limited their outcomes to changes in pain and function over twelve weeks or less. Providing individualised devices has the potential to reduce power and limit generalisability. Additionally, limiting follow-up to the short term may result in unobserved effects and the inability to determine if the effect remains over the longer term. Due to complex nature of chronic nonspecific LBP, measuring other domains that LBP affects, such as the level of physical activity [21], may help to better understand the response to orthotic devices.

Like the effectiveness of foot orthoses for chronic nonspecific LBP, the mechanism of action is similarly inconclusive. While previous research has demonstrated that foot orthoses can alter the kinematics of proximal structures [13], the effects have been found to be small, inconsistent and primarily seen in the foot and tibia rather than extending proximally [22]. So while it is possible that small changes may be significant for the development of pathology, due to the repetitive nature of gait, the lack of a homogenous kinematic effect indicates that alterations to kinematics may not be the primary action of foot orthoses in the treatment of more proximal injuries, such as chronic nonspecific LBP [13].

An alternative mechanism of action of foot orthoses is by influencing muscle activity, with some research demonstrating that this is the case when walking and running [23]. This includes changes to timing and intensity of contraction of muscles of the lower leg and thigh [24], and the superficial muscles of the lower back [25]. However, it is unclear how foot orthoses may have this effect and whether or not it is beneficial [23]. Nevertheless, the potential for proximal muscle activity to be altered with the use of foot orthoses may be significant in the treatment of chronic nonspecific LBP, as this condition is associated with dysfunction of deep trunk muscles such as the transversus abdominis [26], and weakness of hip muscles including gluteus medius [27].

Current evidence regarding the efficacy of foot orthoses for treatment of chronic nonspecific LBP, particularly in the long term, is inconclusive, as is the potential effect of foot orthoses on more proximal hip and trunk muscle function. This randomised controlled trial aims to investigate the effectiveness of prefabricated foot orthoses compared to a placebo intervention on pain and function in people with chronic nonspecific LBP after 12, 26, and 52 weeks. The secondary aims include determining the effect that these interventions have on participants’ gluteus medius muscle activity and transversus abdominis muscle thickness over 12 weeks, and physical activity over 12, 26 and 52 weeks. Additionally, the effectiveness of the interventions on pain and function per foot type (supinated, neutral, or pronated), as well as, intervention adherence and adverse events will also be investigated.

## Methods

### Design

**Fig. 1:**
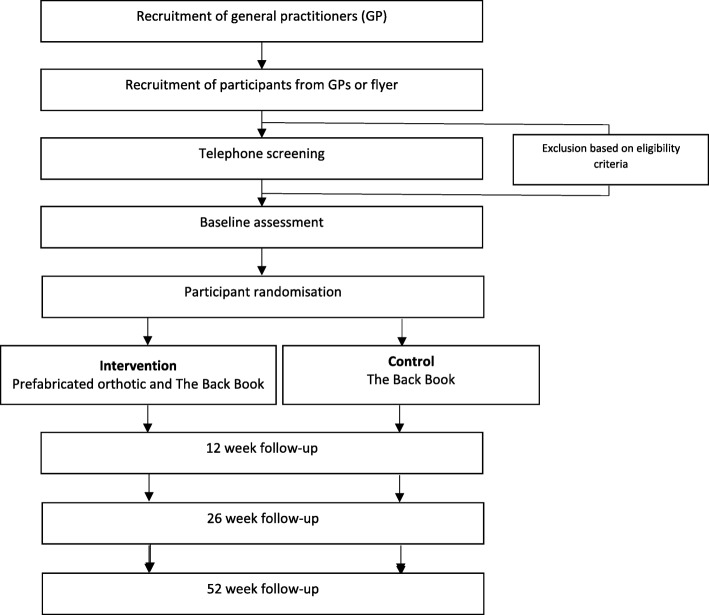
Flow of participants through the trial

This is a two-armed, parallel-group (intervention versus control), participant and assessor blinded superiority randomised (1:1) controlled trial (Fig. 1). Allocation concealment will be used to prevent selection bias by the use of sequentially numbered, opaque sealed envelopes containing a permuted block random allocation schedule with mixed block lengths of four and six participants. The trial will be conducted at the University of Newcastle Ourimbah Campus and the Podiatry Clinic at Wyong Hospital, both located in NSW, Australia. The trial has been prospectively registered on the Australian New Zealand Clinical Trials Registry (ACTRN12618001298202). The results of this trial will be reported using the CONSORT statement [28].

### Ethical approval

Ethical approval has been provided by the University of Newcastle Human Ethics Committee (H-2017-0345). Written informed consent will be obtained from participants before baseline testing.

### Participants

Participants will be aged 18 to 65 years, male, female or of indeterminate sex, proficient in English, have a history of chronic nonspecific LBP as confirmed by a general practitioner (GP), and report a score of 3 or greater for their chronic nonspecific LBP on the numeric pain rating scale to ensure a minimally clinically important change for this score can be measured [29]. The chronic nonspecific LBP will need to be located between the ribs and buttock creases to be eligible [30]. Participants will be excluded if they have significant or worsening signs of neurological deficit, inflammatory joint disease, are pregnant, or have previous (within the last 12 months) or current use of foot orthotic devices.

Potential participants will be recruited using an advertising flyer or by a participating GPs informing their patient of the project. Participants must be seen by a GP prior to commencing of the study so that chronic nonspecific LBP can be diagnosed and their eligibility for the study verified.

GPs in the Central Coast region of NSW will be contacted via phone or in person by one of the researchers prior to participant recruitment. They will be invited to participate in the project as a referring health professional. If they agree to participate, they will be asked to assess and diagnose patients presenting with chronic nonspecific LBP and then screen the patient as per the study’s inclusion and exclusion criteria. Additionally, the advertising flyer will be displayed at health professional clinics in the Central Coast region of NSW and will have the researcher’s contact details on it. Potential participants that see the flyer and firstly contact the researcher will be referred to the University of Newcastle Ourimbah Campus GP to have their LBP assessed and then screened against the study’s inclusion and exclusion criteria for chronic nonspecific LBP. The GP screening participants will provide eligible participants with a general participant information statement. This contains one the researcher’s contact details so that interested participants can contact the researcher to organise baseline testing. The general participant information statement will inform potential participants about the study’s requirements, including that participants will be randomised to one of two groups and receive a conservative (non-surgical and not pharmacological) intervention commonly used by health professionals. It will also advise participants that once they are randomised to a group, they will be given another participant information statement that will provide specific information about their assigned intervention.

### Interventions

Participants in the control group will receive ‘The Back Book’ educational booklet (Additional file 1) which contains written information about techniques for dealing with back pain. It has been demonstrated that this booklet has no significant effect on improving pain or function in people with chronic nonspecific LBP [31].

**Fig. 2:**
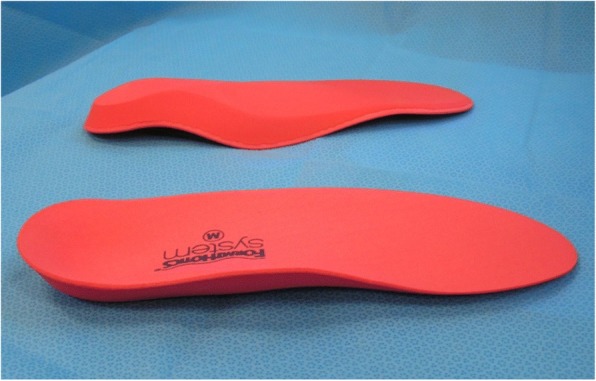
Example of prefabricated orthoses

The intervention group will also receive ‘The Back Book’. In addition, they will be issued a pair of Formthotics™ prefabricated foot orthoses (Foot Science International Ltd., Christchurch, New Zealand, Fig. 2). The prefabricated foot orthoses will be heat moulded to fit the participant’s feet as per the manufacturer’s instructions. The devices will be worn in the participant’s usual footwear for 1 year from baseline. Wearing in instructions will be provided by the therapist (registered podiatrist), issuing the intervention. During the fitting process participants are shown, by the therapist, how the devices are fitted to their footwear. Participants are instructed to wear the devices as much as possible and that they may change them to other suitable shoes. Shoe suitability will be discussed as part of the fitting process. Participants in the prefabricated orthotic group will be provided with a usage diary. This and each intervention will be provided to participants by the therapist who is located in a separate room, after baseline questionnaires and clinical measurements are completed.

### Data collection sessions

Participants will be required to attend two data collection sessions: baseline and 12 weeks later (Fig. 1). Follow-up questionnaires will be mailed to participants at 26 and 52 weeks after the baseline session.

After the GP has screened a participant and they have contacted the researcher for phone screening, a baseline testing session will be arranged, and they will be sent a general health questionnaire which they will be asked to bring completed to the baseline testing session. The general health questionnaire consists of questions about sociodemographic variables. During the phone screening, potential participants will be asked about current and previous treatments for their LBP and instructed to wear suitable attire for clinical testing and to bring multiple pairs of lace-up or Velcro, enclosed footwear.

The assessor (S.S.), who will be blinded to treatment allocation, will conduct baseline and follow-up anthropometric and clinical measurements, and issue self-administered baseline and follow-up questionnaires to participants.

#### Anthropometric measurements

Weight will be measured using standard bathroom scales. Waist circumference will be measured using a tape measure. Height will be measured with a wall-mounted tape measure.

#### Clinical measurements

To avoid an order effect, the order of the clinical measurements for each participant will be randomised using an online random sequence generator. For measures that have multiple components, such as gluteus medius muscle activity and transversus abdominis muscle thickness (Table 1), the order of these individual components will also be randomised. For any clinical measurement conducted at subsequent sessions, the order will be the same as at baseline. The left and right side will be tested for each clinical measurement, except for the gluteus medius and transversus abdominis measurements where only the right side will be measured (Table 1). To comply with the assumption of independence of data, when the left and right sides are measured, both sides will be combined to obtained an average [51]. When both sides are measured (e.g. range of motion), two measurements will be taken per side. All clinical measurements will be taken at baseline with only the gluteus medius muscle activity and transverses abdominis muscle thickness remeasured at the 12 week follow-up.

**Table 1:** Overview of clinical measurements

Clinical measurement	Technique
Weight bearing ankle joint dorsiflexion ROM (knee flexed) [32]	• Participant stands facing the wall and places two hands on the wall shoulder-width apart. The leg being tested is positioned perpendicular to the wall and they extend their ipsilateral hip. • Participant then performs the lunge, dorsiflexing their ankle to its maximum end-point with the participant’s knee flexed. • The foot must remain perpendicular to the wall and their calcaneus must remain on the ground. • The digital inclinometer is then placed on the anterior aspect of their tibia, approximately 15 cm distal to the tibial tuberosity and the angle recorded.
Weight bearing ankle joint dorsiflexion ROM (knee extended) [33]	• As described above but with the knee extended.
Hamstring ROM [34]	• Participant is supine with the hip and ipsilateral knee flexed to 90 degrees. • Assessor extends the knee with the inclinometer, attached to the neoprene material, on the anterior tibia until the end ROM.
Internal and external hip ROM (hip flexed to 90 degrees) [35]	• Participant is positioned in a supine position with their hip and knee at 90 degrees and their lower legs are hanging over the edge of the examination chair. • Assessor rotates hip through internal ROM, holding the lower leg that has the inclinometer attached to the neoprene material, until the end of internal ROM. The inclinometer is positioned on the distal third of the fibula. • The same is then done for external ROM.
Internal and external hip ROM (hip extended to 180 degrees) [35]	• As described above but the participant is lying on their back so that the hip is at 180 degrees. The knee remains flexed at 90 degrees.
Frontal plane ankle joint ROM [36]	• Participants is seated with their hip and knee at 90 degrees. • The goniometer will be placed on the front of their ankle and top of their foot. The goniometer will be positioned at the midpoint between the malleoli and align with the second digit of the foot being tested. • Participants will be instructed to maximally invert and evert their foot, with the change in angle from the most everted to the most inverted recorded.
Lateral flexion ROM of the lumbar spine [37–39]	• Participant stands upright with a hand on the outside of the ipsilateral thigh. • Participant then laterally flexes so that the hand moves distally on the thigh to the end ROM. • The distance from the starting position of the middle finger to its final position will be measured with a tape measure.
Foot posture index (FPI) [40]	• Participants stand in their normal base of gait and take 6–8 steps on the spot. • The assessor will then grade each foot once against the 6 criteria of the FPI and assign a score.
Glutues medius muscle activity [41–45]	• Using the Delsys Trigno electromyographic device, three side-lying hip abduction maximum voluntary isometric contractions (MVIC) for 5 s, with a 60 s rest between contractions, and resistance applied by the examiner at the ankle, will be conducted • Gluteus medius data will be recorded once barefoot and shod on a hard level surface, at the participant’s self-selected comfortable walking speed, for ten seconds (minimum of five strides).
Transversus abdominismuscle thickness [46–50]	• Using the Shenzhen Mindray M5 ultrasound, three Images will be taken at each of the following: rest, during an active straight leg raise (ASLR) on the right to a height of 20 cm, and an abdominal drawing in manoeuvre (ADIM). • At rest and during the ASLR, the participants will be positioned supine with hips and knees flat on the plinth and arms by their side. A supine hook lying position, with hips flexed to 40–60 degrees and knees flexed to 90–100 degrees, will be used for the ADIM. For the ADIM, participants will be instructed to draw their belly button towards their spine. • Three images will be taken for each position and all will be collected at the end of normal exhalation.

**Fig. 3:**
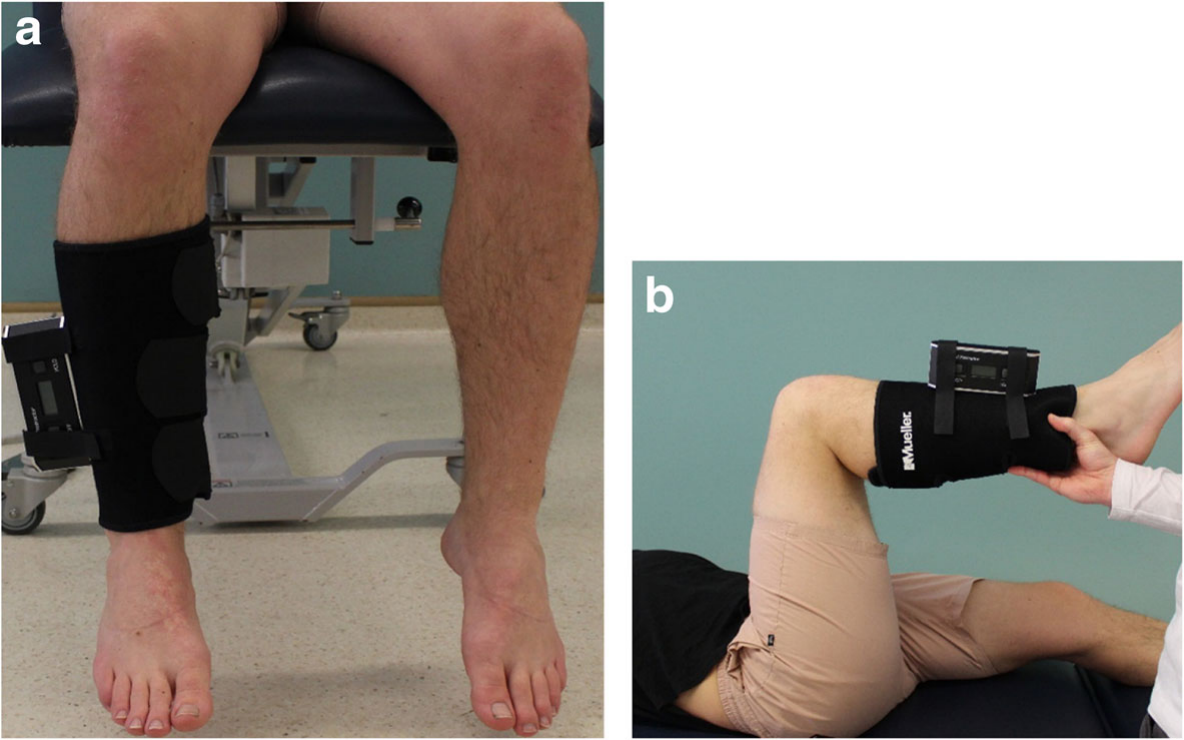
**a** Digital inclinometer position for hip internal and external rotation range of motion. **b** Digital inclinometer position for hamstring range of motion

The ROM measures and foot posture index (FPI) will only be measured at baseline and will help to provide an overview of the participants within this trial. To measure range of motion (ROM) a digital inclinometer (Angle Sensor 82201B-00) will be used except for frontal plane ankle joint ROM and lateral flexion of the lumbar spine, which will be measured with a plastic goniometer and tape measure respectively. The digital inclinometer will be adhered to a piece of neoprene that circumducts the lower leg for internal and external hip and hamstring ROM (Fig. 3).

The FPI will be used to assess foot type (Table 1). The FPI consists of six criteria which are graded on a 5 point scale from − 2 to 2, with the score for each criterion summed to create a total score per foot [40]. Foot type will be categorised according to the following: supinated foot (− 1 to − 12), neutral (0 to 5), and pronated (6 to 12) [52].

Skin over the right gluteus medius muscle will be prepared according to the surface electromyography for the non-invasive assessment of muscles (SENIAM) guidelines and a wireless electromyographic sensor (Delsys Trigno™) adhered parallel to the direction of muscle fibres, half way between the line from the iliac crest and the greater trochanter [41]. Foot switches will be used to measure gait events and will be adhered to the planter posterior aspect of the calcaneus, the plantar aspect of the 1st and 5th metatarsal heads, and the plantar surface of the interphalangeal joint of the hallux. Electromyographic measurement of the gluteus medius muscle has been shown to be reliable [42, 53].

Transversus abdominis muscle thickness will be measured with a Shenzhen Mindray M5 diagnostic ultrasound machine and Mindray linear transducer (7L4s), in B-mode at 7.5 MHz, positioned adjacent and perpendicular to the abdominal wall, 25 mm antero-medial to the midpoint between the ribs and ilium, on the mid-axillary line and parallel to the transversus abdominis muscle fibres. Thickness measurements will be made at the hyperechoic fascial lines between the superficial and deep edges of the muscle. Ultrasound measurement of the transversus abdominis muscle has been shown to be reliable [46].

### Follow-up sessions (12, 26 ad 52 weeks)

Participants will be required to return 12 weeks after the baseline testing session to have their gluteus medius muscle activity and transversus abdominis muscle thickness remeasured. Participants will also be required to complete the numeric pain rating scale, Oswestry Disability Index, and International Physical Activity Questionnaire (IPAQ-7). Participants will also be mailed these questionnaires 26 and 52 weeks after baseline testing. At the 12 week follow-up, participants will be asked if they have read ‘The Back Book’. The participant information statements contain the researchers’ contact details so participants can report adverse events at any stage of the trial. Adverse events will be managed by the researchers or participants will be referred to their GP.

### Primary outcomes

The primary outcomes will be change in pain and function from baseline to 12, 26 and 52 weeks. The primary follow-up time point is 12 weeks. It is important to highlight that two primary outcome measures will be used because they are both of clinical importance.

#### Primary outcome measurements

Pain will be assessed using the numeric pain rating scale which is an 11-point scale from zero to ten with zero being ‘no pain’ and 10 ‘the most intense pain imaginable’ [29]. Participants select a value that is most in line with the intensity of pain that they have experienced in the last 24 h. The minimal clinically important difference has been demonstrated to be two out of 10 points [29].

Function will be assessed using the Oswestry Disability Index which consists of ten sections related to activities of daily living commonly affected by LBP [54]. Each section is scored from zero to five with the sum of the scores presented as a percentage. Higher values indicate a more severe impact of LBP on daily living. The minimal clinically important difference has been reported to be 12.88 [55].

### Secondary outcomes

The secondary outcomes will include:

(i) Gluteus medius muscle activity from baseline to 12 weeks follow-up;
(ii) Transversus abdominis muscle thickness from baseline to 12 weeks follow-up;
(iii) correlation between foot type and change in pain and function;
(iv) physical activity from baseline to 12, 26, and 52 weeks follow-up;
(v) number of hours per day and week the prefabricated orthotic is worn over 12 weeks; and
(vi) self-reported adverse events associated with the interventions over 52 weeks.

#### Secondary outcome measurements

Gluteus medius muscle activity, transversus abdominis muscle thickness and foot type will be assessed as outlined in Table 1. Physical activity will be assessed using the IPAQ-7 which consists of seven questions about the amount and intensity of moderate and vigorous physical activity undertaken in the past 7 days, as well as, the amount of time spent sitting [56]. The diary provided to participants in the prefabricated foot orthotic group will be used by participants to record the number of hours per day they have worn the orthotic device over 12 weeks. In addition to participants being able to contact the researchers at any stage of the trial to report adverse events, they will be asked if such events have occurred at the face to face follow-up session at 12 weeks.

### Sample size

The sample size for this trial was calculated to find minimal clinically important change of 2 (s = 2.5) on the numeric pain rating scale [29] with 80% power, a 5% Type I error rate and non-adherence rate of 20%. Numeric pain rating scale was chosen for the calculation instead of the Oswestry Disability Index because it had the smaller effect size and thus would require the larger sample size. The calculation adjusted for non-adherence required a total sample size of 30 per group, therefore a total of 60 participants will be recruited.

### Statistical and data analysis

Statistical analysis will be conducted using the latest version of appropriate statistical software (e.g. SPSS or STATA). Analyses involving primary outcomes will be conducted on an intention-to-treat principle using all randomised participants. Missing data will be replaced using multiple imputation. Normality will be assessed using the Shapiro Wilks test. For the primary outcomes of pain and function (numeric pain rating scale and Oswestry Disability Index), data will be compared using analysis of covariance (ANCOVA) to investigate the change from baseline to each of the follow-up points. The baseline measure will be the only covariate used in each analysis.

The IPAQ-7 will be used to determine changes in physical activity from baseline for participants using analysis of covariance at each time point (12, 26 and 52 weeks). The correlation between IPAQ-7 score and change in primary outcomes will be investigated and where appropriate (significant correlations identified) a regression analysis performed. For the secondary outcomes of gluteus medius activity and transversus abdominis thickness, ANCOVA will be used to investigate change from baseline following 12 weeks of the intervention for each variable. The baseline measure will be the only covariate used in each analysis. Cohen’s d will used to calculate effect sizes for both primary and secondary outcomes.

Pearson correlation coefficients will be performed between foot type and the change in primary outcome variables at 12, 26, and 52 weeks. Where significant correlations are found, a regression analysis will then be performed to determine the ability of foot type to predict treatment outcomes.

## Discussion

This randomised controlled trial will be undertaken to determine the effectiveness of prefabricated foot orthoses for reducing pain and improving function in people with chronic nonspecific LBP over 12, 26, and 52 weeks. Previous research investigating the effectiveness of foot orthotic devices for the treatment of chronic nonspecific LBP has generally been of moderate methodological quality and high heterogeneity [16]. Our study attempts to build on previous research by using a prefabricated foot orthotic, measuring proximal muscle response to the intervention, intending to recruit different foot types, assessing the impact of the intervention on additional domains, and conducting the trial over 52 weeks.

Prefabricated foot orthotic devices have been chosen, compared to custom orthotic devices, because they are commonly prescribed by a range of health professionals and are relatively inexpensive. Coupled with the community based population that will be recruited, the orthotic devices will not require modification, therefore increasing the generalisability of the results. Additionally, the prefabricated orthoses chosen have been widely used in previous research with minimal adverse events reported [57, 58].

Measurement of proximal muscle response to the intervention will help determine if foot orthoses induce changes in muscle function over 12 weeks. It is possible that this is a potential mechanism for any therapeutic effect of foot orthoses for chronic nonspecific LBP that may be demonstrated by this trial. Previous research on the potential mechanism of action of foot orthoses has tended to focus on kinematic and shock attenuation paradigms, with findings from meta-analyses indicating small and often clinically insignificant effects [59]. Fewer studies have focused on the neuromuscular paradigm, limiting pooled analyses. However, of the trials that have investigated the neuromuscular response to foot orthoses, the focus has largely been on the muscles in the lower leg [59]. Of these trials, the effect of a range of different foot orthotic designs has been evaluated, with the consistent outcome being increases in muscle amplitude in response to the orthotic [23, 59]. Similar increases in gluteus medius and transversus abdominis muscle activity have been found in response to foot orthoses however these trials have included fewer than twenty participants, investigated the immediate effect of the devices, and included people without chronic nonspecific LBP [25, 60, 61]. We plan to build upon previous research by evaluating the effect that prefabricated foot orthoses have on gluteus medius activity and transversus abdominis muscle thickness in sixty people with chronic nonspecific LBP over 12 weeks.

Previous research investigating foot orthotic devices for a range of other musculoskeletal conditions have used flat insoles in the control group [57, 62, 63]. However, flat insoles may have some therapeutic or biomechanical effect and are therefore more appropriately referred to as a sham intervention [64]. Additionally, some participants with chronic nonspecific LBP may benefit from the potential cushioning of a flat insert (for example those with a pes cavus foot type). Therefore the authors believe that an education-based intervention, ‘The Back Book’, is better suited to act as a placebo intervention because it has been shown to be ineffective for reducing pain and function in people with chronic nonspecific LBP [31].

It is also anticipated that this trial will help develop an understanding of the proportions of foot types in people with chronic nonspecific LBP, so that future studies can be adequately powered for the interaction between foot type and foot orthoses. Previous research has either not defined participant’s foot type [16], or has focused only on pronated feet [19]. Castro-Mendez and colleagues [19] found a significant effect in favour of foot orthoses for reducing pain in those with chronic nonspecific LBP and pronated feet, however the effect in relation to other foot types is unknown.

This protocol is based on the SPIRIT guidelines [65]. However, given that this trial will use a placebo intervention for the control group, sources of bias associated with this type of design are important to consider. We will attempt to control ascertainment or detection bias [66], by having two levels of participant information statements and consent forms and maintaining allocation concealment. Nevertheless, some participants such as those that know each other, may discuss their interventions outside of the trial and therefore discover the differences. Given that this trial is designed to have a placebo intervention, a ‘no-treatment’ group was not used because of the potential for participants’ results or behaviour to be influenced by resentful demoralisation [66]; however, this may still occur if participants become aware of the different interventions.

Therefore, whilst we have designed the trial to maintain methodological rigour and control for potential sources of bias associated with a placebo control group, there are still some potential limitations. We will not be able to account for a potential placebo effect or prevent participants from undergoing other treatments external to the study, both of which may confound any treatment effects. We will record any external treatment participants undertake to help with the interpretation of the results.

This randomised controlled trial will primarily investigate the effectiveness of prefabricated foot orthoses for reducing pain and improving function in people with chronic nonspecific LBP over 52 weeks. Additionally, this study will investigate the effect that prefabricated foot orthoses have on gluteus medius muscle activity and transversus abdominis muscle thickness over 12 weeks, as well as, on the level of physical activity over 52 weeks. The influence that foot type has on the primary outcomes will also be investigated as a secondary outcome. This trial is expected to help inform clinicians and researchers about the effectiveness of prefabricated orthotic devices for the treatment of chronic nonspecific LBP, as well as, help explain their potential mechanism of action in this population.

## Additional file

Additional file 1: The Back Book. (PDF 531 kb)

## Abbreviations

ANCOVA: Analysis of covariance
FPI: Foot posture index
GP: General practitioner
IPAQ-7: International Physical Activity Questionnaire
LBP: Low back pain
ROM: Range of motion
SENIAM: Surface electromyography for the non-invasive assessment of muscles
