# Supplementary material for: Prefabricated foot orthoses compared to a placebo intervention for the treatment of chronic nonspecific low back pain: a study protocol for a randomised controlled trial
Source: J Foot Ankle Res. 2018 Oct 16;11:56. doi: 10.1186/s13047-018-0299-5 (PMC6192308; doi:10.1186/s13047-018-0299-5)
Supplement: Supplementary file 1 — The Back Book. (PDF 531 kb) [file 13047_2018_299_MOESM1_ESM.pdf]

# THE BACK BOOK

© TSO 2002
Applications for reproduction should be made to TSO, St Crispins, Duke Street, Norwich NR3 1PD

Web pdf version 13/6/03 11:52 AM Page ii

© TSO 2002

Applications for reproduction should be made to TSO, St Crispins, Duke Street, Norwich NR3 1PD

The Publisher is not responsible for any statement made in this publication. Advice is offered by the authors for information only, and is not intended for use without independent substantiating investigation on the part of the potential users.

First published 1996
Second edition published 2002

Single copies ISBN 0117029491 Price £1.25

**Discounts apply for larger quantities. Call 0870 600 5522**

Pack of 10 copies ISBN 0117029505

| | |
|---|---|
| 1-5 packs | Price £12 per pack |
| 6-19 packs | Price £10 per pack |
| Over 20 packs | Price £6 per pack |

Also available from TSO

- 'Get Back Active' (ISBN 0117029408) a video based on the Back Book giving practical guidance on handling back pain
- 'The Whiplash Book' (ISBN 011702029X) offering advice on how to deal with a whiplash injury - based on the latest research

The help and advice from colleagues too numerous to mention is gratefully acknowledged.

Printed in the United Kingdom for The Stationery Office

© TSO 2002
Applications for reproduction should be made to TSO, St Crispins, Duke Street, Norwich NR3 1PD

# THE NEW APPROACH TO BACK PAIN

Back pain is very common and causes a great deal of misery but, fortunately, serious or permanent damage is rare. There has been a revolution in thinking about back care and we now deal with it in a different way. This booklet sets out the facts and shows you how to get better as quickly as possible. It's based on the latest research.

What you do about back pain yourself is usually more important than the exact diagnosis or treatment.

An attack of back pain can be alarming. Even a minor back strain can be very painful and it's natural to think that something dreadful might have happened. But stop and look at the facts:

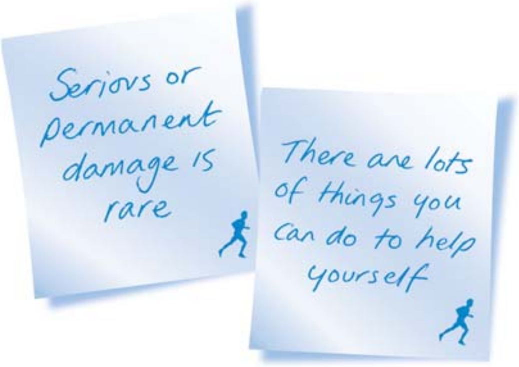




© TSO 2002
Applications for reproduction should be made to TSO, St Crispins, Duke Street, Norwich NR3 1PD

Web pdf version 13/6/03 11:52 AM Page 2

# BACK FACTS

- Most back pain is not due to any serious disease.
- The acute pain usually improves within days or a few weeks, at least enough to get on with your life. The long-term outlook is good.
- Sometimes aches and pains can last for quite a long time. But that doesn't mean it's serious. It does usually settle eventually - even though it's frustrating that no one can predict exactly when! Most people can get going quite quickly, even while they still have some pain.
- About half the people who get backache will get it again within a couple of years. But that still does not mean it's serious. Between attacks most people return to normal activities with little if any pain.
- What you do in the early stages is very important. Rest for more than a day or two usually does not help and may actually prolong pain and disability.
- Your back is designed for movement: it needs movement - a lot of movement. The sooner you get moving and doing your ordinary activities as normally as possible, the sooner you will feel better.
- The people who cope best with back pain are those who stay active and get on with life despite the pain.



© TSO 2002
Applications for reproduction should be made to TSO, St Crispins, Duke Street, Norwich NR3 1PD

# CAUSES OF BACK PAIN

Your spine is one of the strongest parts of your body. It is made of solid bony blocks joined by discs to give it strength and flexibility. It is reinforced by strong ligaments, and surrounded by large and powerful muscles that protect it. Most simple back strains do not cause any lasting damage.

## Despite what you might have heard:

- Only a few people with back pain have a slipped disc or a trapped nerve. Even then, it usually gets better by itself. Very few back problems ever need surgery.
- X-rays and MRI scans can detect serious spinal injuries, but they don't usually help in ordinary back pain. They may even be misleading. Doctors sometimes mention 'degeneration' which sounds frightening, but it's not damage or arthritis. These are the normal changes with age - just like grey hair.

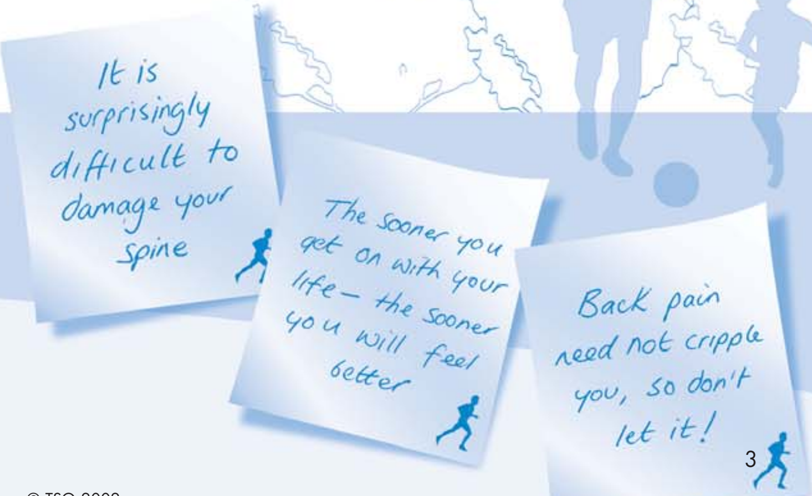




© TSO 2002
Applications for reproduction should be made to TSO, St Crispins, Duke Street, Norwich NR3 1PD

- Your doctor or therapist will often not be able to pinpoint the source of the pain. Again, it's frustrating not to know exactly what is wrong. Actually, in another way it's good news - you do not have any serious damage to your spine.

Most back pain comes from the working parts of your back - the muscles, ligaments, and small joints. Your back is simply not moving and working as it should. You can think of it being 'out of condition'. So what you should do is get your back moving and working properly again. This stimulates its natural ability to recover.

## REST OR STAY ACTIVE?

The old fashioned treatment for back pain was rest. Some people with back pain were sent to bed for weeks or even months on end, just waiting for the pain to disappear. We now know that bed rest for more than a day or two is the worst possible treatment, because in the long term it actually prolongs the pain:

- You get stiff.
- Your muscles get weak.
- Your bones get weaker.
- You lose physical fitness.
- You get depressed.
- The pain feels worse.
- It is harder and harder to get going again.

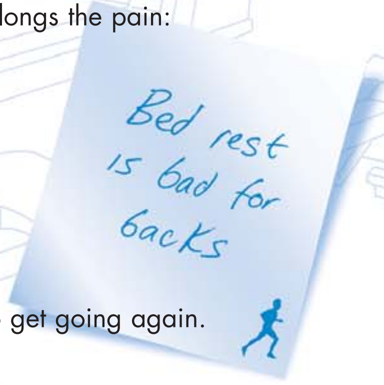




© TSO 2002
Applications for reproduction should be made to TSO, St Crispins, Duke Street, Norwich NR3 1PD

No wonder it didn't work! We no longer use bed rest to treat any other common condition and it's time to stop bed rest for back pain.
You may be limited in how much you can do when the pain is bad. You might even be forced to stay in bed at the start. But only for a day or two. Bed rest is not a treatment – it's simply a short-term consequence of the pain. The most important thing is to get moving again as soon as you can.

## ACTIVITY IS GOOD

Your whole body must keep active to stay healthy. It thrives on use.

*Regular physical activity:*

- Develops your muscles.
- Keeps you supple.
- Gives you stronger bones.
- Makes you fit.
- Makes you feel good.
- Releases natural chemicals that reduce the pain.

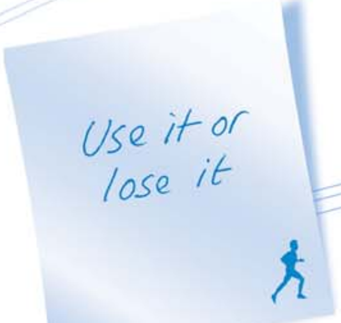




© TSO 2002
Applications for reproduction should be made to TSO, St Crispins, Duke Street, Norwich NR3 1PD

Even when your back is painful, you can make a start without putting too much stress on it.

- Walking
- Swimming
- Exercise bike
- Dancing/yoga/keep fit
- In fact, most daily activities and hobbies.

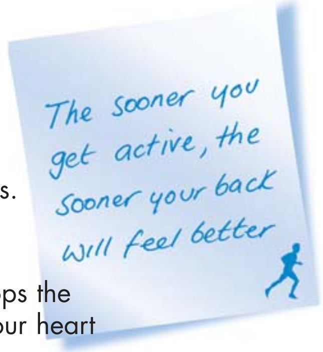


Exercise gets your back moving again by stretching tight muscles and joints, and stops the working parts seizing up. It also makes your heart and lungs work and improves physical fitness.

Different things suit different people. Experiment - find what works best for you and your back. Your goal is to get moving and steadily increase your level of activity. Do a little bit more each day.

Getting stiff joints and muscles working can be painful. Athletes accept that when they start training, their muscles can hurt and they have to work through the pain barrier. But that does not mean they are doing any damage. So don't worry if exercise makes you a bit sore at first – that's usually a sign you are actually making progress! As you get fully fit the pain should ease off.

No-one pretends it's easy. Pain killers and other treatments can help to control the pain to let you get started, but you still have to do the work. There is no other way. You have a straight choice: rest and get worse, or get active and recover.



© TSO 2002
Applications for reproduction should be made to TSO, St Crispins, Duke Street, Norwich NR3 1PD

Do not fall into the trap of thinking it will be easier in a week or two, next month, next year. It won't! The longer you put it off, the harder it will be to get going again. The faster you get back to normal activities and back to work the better - even if you still have some restrictions.

## DEALING WITH AN ATTACK OF BACK PAIN

Most people manage to deal with most attacks themselves. What you do depends on how bad your back feels. However, because there's no serious damage, you can usually:

- Use something to control the pain.
- Modify your activities for a time, if necessary.
- Stay active and get on with your life.

Some people have more persistent pain – but the same principles apply.

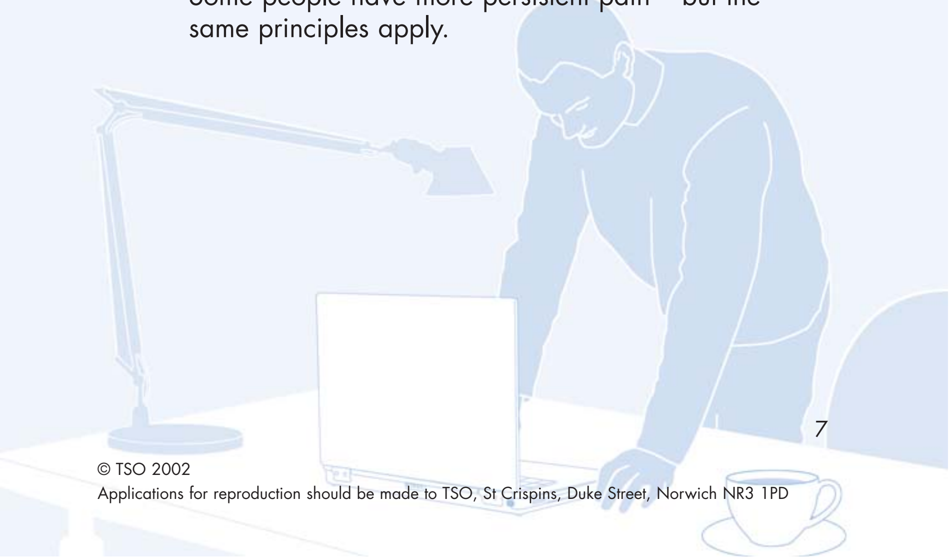



© TSO 2002
Applications for reproduction should be made to TSO, St Crispins, Duke Street, Norwich NR3 1PD

Web pdf version 13/6/03 11:52 AM Page 8

## Control of pain

There are many treatments which can help - even if there is no miracle cure. They may not remove the pain completely, but they should control it enough to let you get active and so make yourself better.

### *Pain killers*

You should not hesitate to use painkillers if you need them. You can safely mask the pain to get active: your body will not let you do any harm. Paracetamol is the simplest and safest pain killer. Or you can use anti-inflammatory tablets like Ibuprofen.

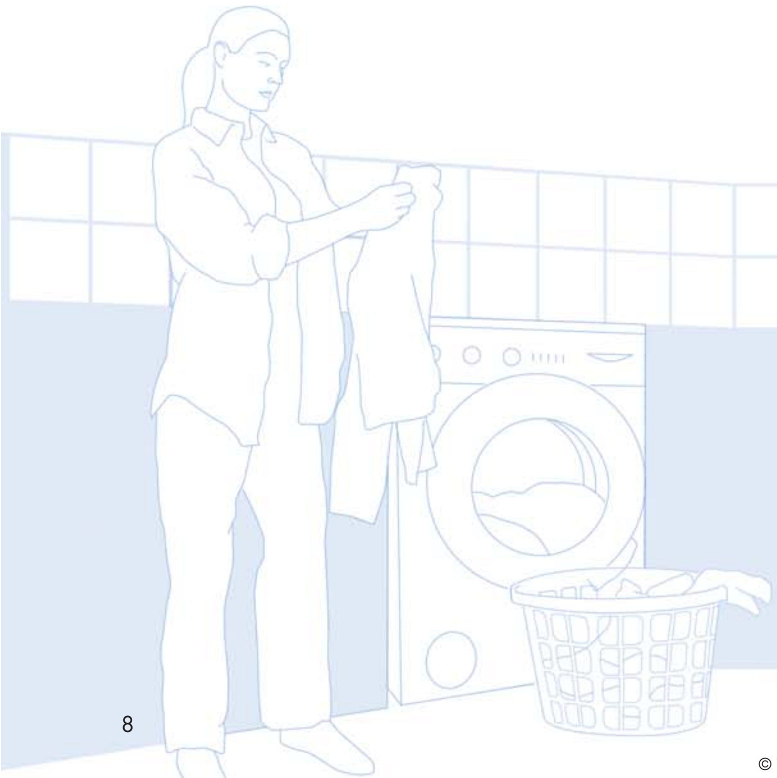



© TSO 2002
Applications for reproduction should be made to TSO, St Crispins, Duke Street, Norwich NR3 1PD

It may surprise you, but these simple over-the-counter painkillers are often the most effective for back pain. The problem is that many people do not use them properly. You should take the full recommended dose and take them regularly every 4-6 hours - do not wait till your pain is out of control. You should usually take the painkillers for a few days, but you may need to take them for a week or two. Few people require anything stronger.

Do not take Ibuprofen or Aspirin if you are pregnant or if you have asthma, indigestion, or an ulcer.

### *Heat & cold*

Heat or cold can be used for short-term relief of pain and to relax muscle tension. In the first 48 hours you can try a cold pack on the sore area for 5-10 minutes at a time - a bag of frozen peas wrapped in a damp towel. Other people prefer heat - a hot water bottle, a bath, or a shower.

### *Massage*

Massage is one of the oldest treatments for back pain. Many people find gentle rubbing eases the pain and relaxes muscle spasm.



© TSO 2002
Applications for reproduction should be made to TSO, St Crispins, Duke Street, Norwich NR3 1PD

Web pdf version 13/6/03 11:52 AM Page 10

*Manipulation*

Most doctors now agree that manipulation can help back pain. It is safe if done by a qualified professional: osteopaths, chiropractors, some physiotherapists and a few doctors with special training. You should begin to feel the benefit within a few sessions and it's not a good idea to have treatment for months on end.

*Other treatments*

Many other treatments such as electro-therapy machines, acupuncture, or alternative medicine are used for back pain and some people feel they help. But be realistic. Despite the claims, these treatments rarely provide a quick fix. Once again, you should feel any benefit quite quickly and there is no value in treatment for months on end. What really matters is whether they help you get active.

## Anxiety, stress and muscle tension

Anxiety and stress can increase the amount of pain we feel. Tension can cause muscle spasm and the muscles themselves can become painful.

Many people get anxious about back pain, especially if it doesn't get better as fast as they expect. You may get conflicting advice - from your family and friends or even from doctors and therapists - which may make you uncertain what best to do. Trust the advice in this booklet - it comes from the latest research. Remember, serious damage is rare and the long-term outlook is good. So do not let fear and worry hold back your recovery.

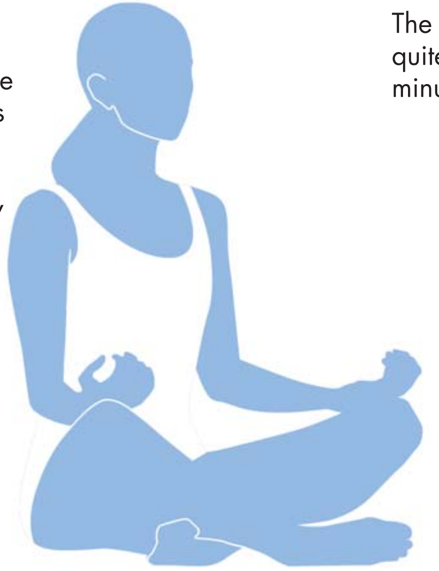



© TSO 2002
Applications for reproduction should be made to TSO, St Crispins, Duke Street, Norwich NR3 1PD

Stress can aggravate or prolong pain. If stress is a problem you need to recognise it at an early stage and try to do something about it. You cannot always avoid stress, but you can learn to reduce its effects by controlled breathing, muscle relaxation and mental calming techniques. One of the best ways of reducing stress and tension is exercise.

*The Swedish relaxation exercise:*

1 Don't try too hard to relax.
2 Find a comfortable position, sitting or lying down – somewhere quiet.
3 Take deep breaths 'slow & steady'; hold for about 15-20 seconds and exhale.
4 Focus your mind on something calm and repetitive.
5 'Let go' when exhaling,. Imagine and concentrate on breathing - not on relaxing.

The 'relaxation response' can sometimes be achieved quite quickly, but deep relaxation may take 10-15 minutes.

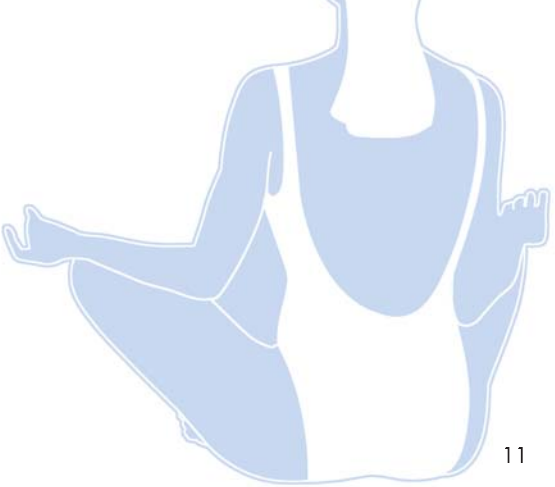



© TSO 2002
Applications for reproduction should be made to TSO, St Crispins, Duke Street, Norwich NR3 1PD

Web pdf version 13/6/03 11:52 AM Page 12

# THE RISK OF CHRONIC PAIN

There has been a lot of research in recent years to identify people at risk of long-term pain and disability. What may surprise you is that most of the warning signs are about what people feel and do, rather than medical findings.

Signs of people at risk of long-term pain:

- Believing that you have a serious injury or damage. Being unable to accept reassurance.
- Believing that hurt means harm and that you will become disabled.
- Avoiding movement or activity due to fear of doing damage.
- Continued rest and inactivity instead of getting on with your life.
- Waiting for someone to fix it rather than believing that you can help yourself recover.
- Becoming withdrawn and depressed.

This all develops gradually and you may not even notice. That’s why it is so important to get going as soon as possible before you develop chronic pain. If you - or your family and friends - spot some of these early warning signs, you need to do something about it. Now, before it is too late. Use the advice in this booklet to work out what you can do to change direction and get on with your life. If you need extra help to get going, you should ask your doctor or therapist.



© TSO 2002
Applications for reproduction should be made to TSO, St Crispins, Duke Street, Norwich NR3 1PD

You may meet a practical problem here. Doctors and therapists deal best with clear-cut diseases and injuries for which they have a cure. We are often not so good at dealing with more ordinary symptoms like back pain. For example, it's no good staying off work and doing nothing for weeks on end to attend therapy. Or waiting months for a surgeon to tell you that you don’t need an operation. That simply delays your recovery! Which is why it really does depend on what you do yourself. You have to make it clear to your doctor or therapist that you realise all this, and what you want is help to get on with your life.

If you are still off work after about a month, you are at risk of developing long-term problems. There is then a 10% risk you will still be off work in a year's time. You could even lose your job. Long before you get to that stage you really need to face up to the problem and take urgent action.

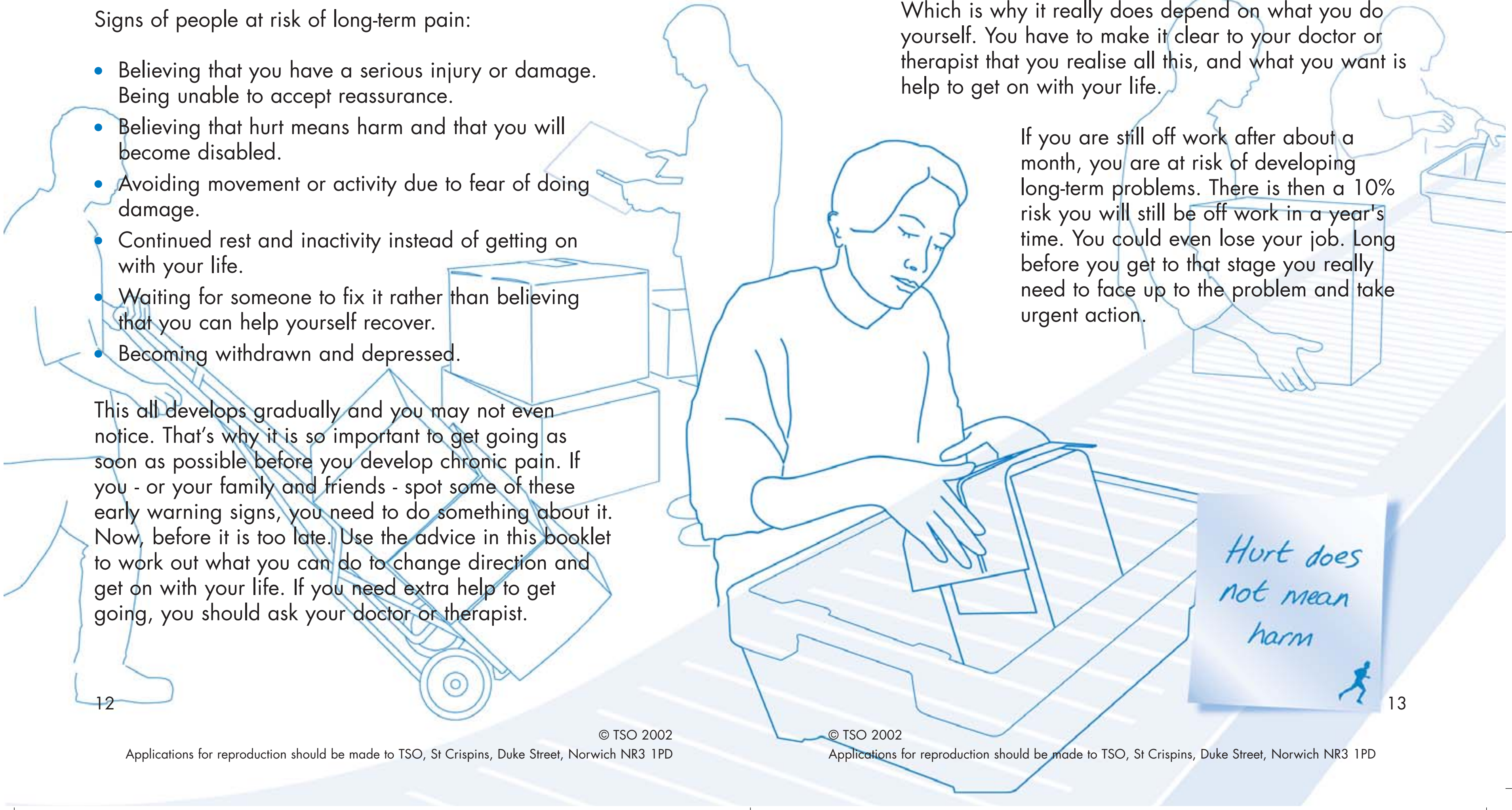




© TSO 2002
Applications for reproduction should be made to TSO, St Crispins, Duke Street, Norwich NR3 1PD

# HOW TO STAY ACTIVE

As we've explained, the sooner you start getting mobile and active again the better. Only if the pain is particularly severe do you need to rest up or be off work. But even then you can still do most daily activities if you think about them first. Work out a plan. What are the problems and how can you get round them? Can you do things a different way?

Try to strike a balance between being as active as you can and not putting too much strain on your back. The basic rules are simple:

- Keep moving.
- Do not stay in one position for too long.
- Move about before you stiffen up.
- Move a little further and faster each day.
- Don't stop doing things - just change the way you do them.

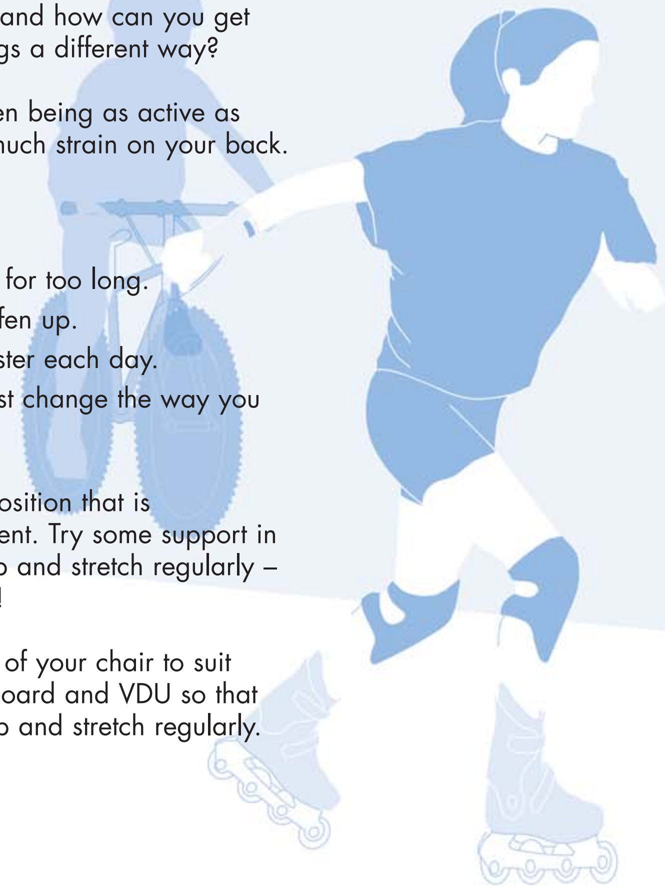

*Sitting* – Chose a chair and position that is comfortable for you - experiment. Try some support in the small of your back. Get up and stretch regularly – take advantage of TV adverts!

*Desk work* – Adjust the height of your chair to suit your desk. Arrange your keyboard and VDU so that you don't feel strained. Get up and stretch regularly.

*Driving* – Adjust your seat from time to time. Try some support in the small of your back. Stop regularly for a few minutes break - get out of the car, walk about and stretch.

*Lifting* – Think before you lift. Don't lift more than you need to. Keep the load close to your body. Don't twist while you are lifting but turn with your feet.

*Carrying and shopping* – Think if you need to carry at all. Carry things hugged to your body or split the load between both hands. Don't carry further than you need to. Use wheels!

*Daily activities/hobbies* – Don't do one thing for too long. Keep changing activities.

*Sports* – Continuing with your normal sport is fine, but you may need to reduce the intensity. Swimming is good - try varying your stroke - backstroke, side stroke, crawl.

*Sleeping* – Some people find a firmer mattress helps - or you can try a sheet of chipboard beneath the mattress. Experiment. Try painkillers an hour before you go to bed.

*Sex* – Fine! – but you may need to try different positions.



© TSO 2002
Applications for reproduction should be made to TSO, St Crispins, Duke Street, Norwich NR3 1PD



© TSO 2002
Applications for reproduction should be made to TSO, St Crispins, Duke Street, Norwich NR3 1PD

# Getting on with your life

It is important to maintain the momentum of your life - and that includes staying at work if you possibly can. Doing things will distract you from the pain, and your back will usually not get any worse at work than it will at home. If you have a heavy job, you may need some help from your work mates. Simple changes may make your job easier.

If you are seeing a doctor or therapist, tell them about your work. Talk to your supervisor or boss if you need to. Tell them about any parts of your job that may be difficult to begin with, but stress that you want to be at work. Offer your own suggestions about how to overcome these problems - you might even show them this booklet.

If you do have to stay off work, it helps to get back as soon as possible – usually within days or a couple of weeks - and even if you still have some pain. The longer you are inactive and off work the more likely you are to develop long-term pain and disability.

If you are not at work within about a month you really should be planning with your doctor, therapist and employer how and when you can return. If you have an occupational health department or health and safety rep, they may be able to assist. Temporary modification to your job or pattern of work may help you get back sooner.



© TSO 2002
Applications for reproduction should be made to TSO, St Crispins, Duke Street, Norwich NR3 1PD

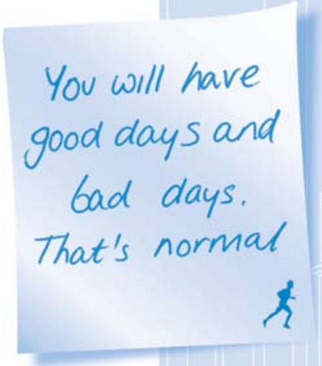


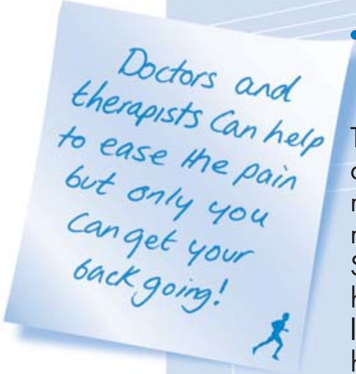


## What doctors can and can't do

Although we have stressed that you can deal with most back pain yourself, there may be times you are uncertain and feel the need to check. That's quite reasonable. But remember there is no quick fix for back pain. So you should be realistic about what you expect from a doctor or therapist.

They can:

- Make sure you don't have any serious disease and reassure you.
- Suggest various treatments to help control your pain.
- Advise you on how you can best deal with the pain and get on with your life.

Try to accept that reassurance and don't let needless worry delay your recovery. You have to share responsibility for your own progress. Some doctors and therapists may be hesitant about handing over and letting you take control. You may have to tell them straight out this really is what you want.

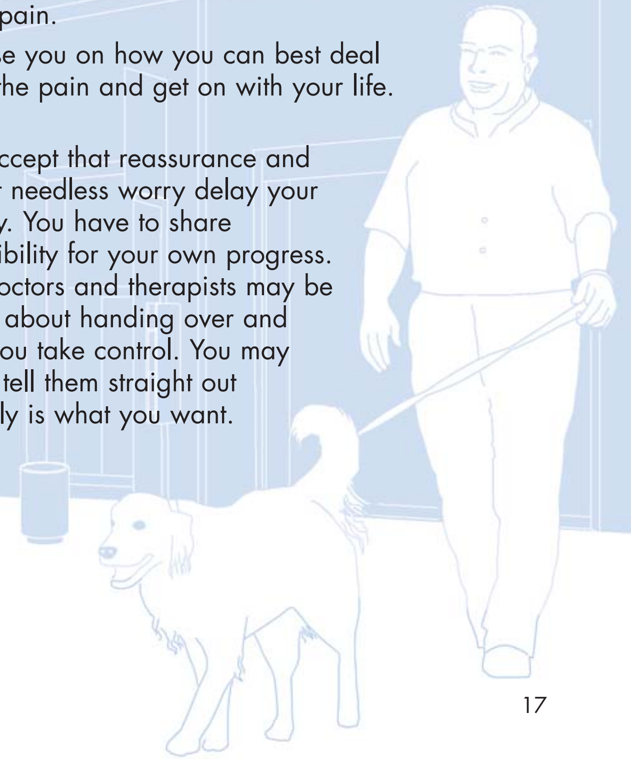



© TSO 2002
Applications for reproduction should be made to TSO, St Crispins, Duke Street, Norwich NR3 1PD

## Warning signs

If you have severe pain which gets worse over several weeks instead of better, or if you are unwell with back pain, you should see your doctor.

Here are a few symptoms, which are all very rare, but if you do have back pain and suddenly develop any of these you should see a doctor straight away.

- Difficulty passing or controlling urine.
- Numbness around your back passage or genitals.
- Numbness, pins and needles, or weakness in both legs.
- Unsteadiness on your feet.

Don't let that list worry you too much.

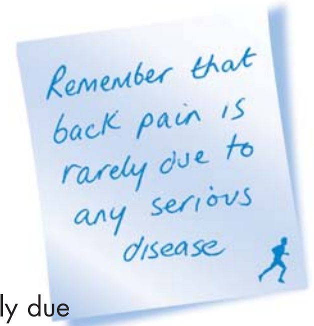


# IT'S YOUR BACK

We've shown you that back pain is rarely due to anything serious and it should not cripple you unless you let it. You've got the facts and the most up-to-date advice about how to deal with back pain. The important thing now is for you to get on with your life. How your back affects you depends on how you react to the pain and what you do about it yourself.

There is no instant answer. You will have your ups and downs for a while - that's normal. But look at it this way:



© TSO 2002
Applications for reproduction should be made to TSO, St Crispins, Duke Street, Norwich NR3 1PD

*There are two types of sufferer*

One who avoids activity ☹
and one who copes ☺

☹ The avoider gets frightened by the pain and worries about the future.

- The avoider is afraid that hurting always means further damage - it doesn't.
- The avoider rests a lot, and just waits for the pain to get better.

☺ The coper knows that the pain will get better and does not fear the future.

- The coper carries on as normally as possible.
- The coper deals with the pain by being positive, staying active and getting on with life.

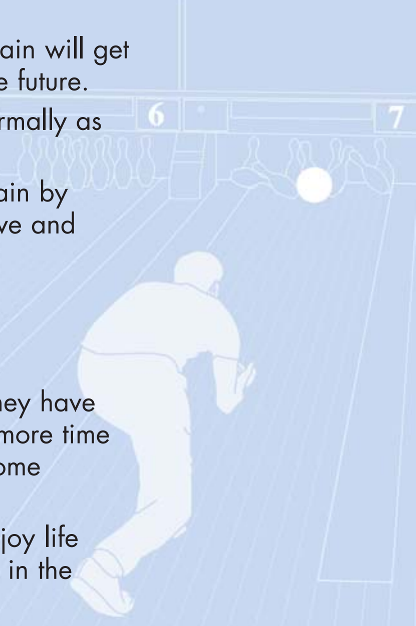


*Who suffers most?*

☹ Avoiders suffer the most. They have pain for longer, they have more time off work and they can become disabled.

☺ Copers get better faster, enjoy life more and have less trouble in the long run.



© TSO 2002
Applications for reproduction should be made to TSO, St Crispins, Duke Street, Norwich NR3 1PD

## So how do I become a Coper and prevent unnecessary suffering?

Follow these guidelines - you really can help yourself.

☺ Live life as normally as possible. This is much better than giving in to the pain.

- Keep up daily activities - they will not cause damage. Just avoid really heavy things.
- Try to stay fit - walking, cycling or swimming will exercise your back and should make you feel better. And continue even after your back feels better.
- Start gradually and do a little more each day so you can see the progress you are making.
- Either stay at work or go back to work as soon as possible. If necessary, ask if you can get lighter or modified duties for a week or two.
- Keep going. It's normal to get aches or twinges for a time.

☹ Don’t rely on painkillers alone. Stay positive and take control of the pain yourself.

- Don’t stay at home or give up doing things you enjoy.
- Don’t get frightened. Continuing pain does not mean you are going to become an invalid.
- Don’t listen to other people’s horror stories.
- Don’t get gloomy on the down days.

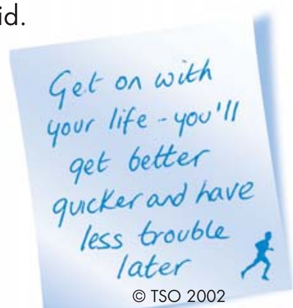




© TSO 2002
Applications for reproduction should be made to TSO, St Crispins, Duke Street, Norwich NR3 1PD

## Remember:

- Back pain is common but it is rarely due to any serious disease. The long-term outlook is good.
- Even when it is very painful that usually doesn’t mean there’s any serious damage to your back. Hurt does not mean harm.
- Bed rest for more than a day or two is usually bad for you.
- Staying active will help you get better faster and prevent more back trouble.
- The sooner you get going, the faster you will get better.
- If you don’t manage to get back to most normal activities quite quickly, you should seek additional help.
- Regular exercise and staying fit helps your general health and your back.
- You have to get on with your life. Don’t let your back take over.

*That's the message from the latest research - you really can help yourself*

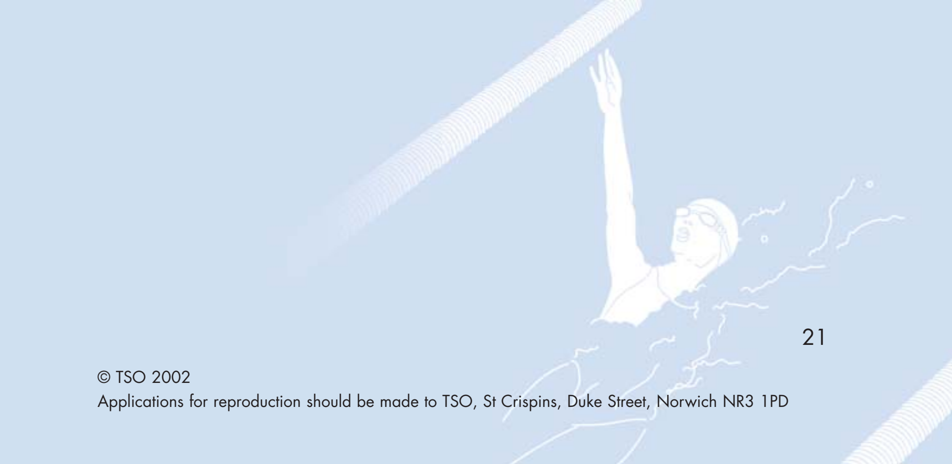



© TSO 2002
Applications for reproduction should be made to TSO, St Crispins, Duke Street, Norwich NR3 1PD

© TSO 2002
Applications for reproduction should be made to TSO, St Crispins, Duke Street, Norwich NR3 1PD
